# Supplementary material for: Combined effects of ambient temperature and food availability on induced innate immune response of a fruit-eating bat (Carollia perspicillata)
Source: PLoS One. 2024 May 24;19(5):e0301083. doi: 10.1371/journal.pone.0301083 (PMC11125493; doi:10.1371/journal.pone.0301083)
Supplement: S1 Fig — Body mass changes was assessed in relative terms as: body mass change (ΔMb) = (mean body mass after injections–mean body mass before injections) / (mean body mass before injections). (PDF) [file pone.0301083.s001.pdf]

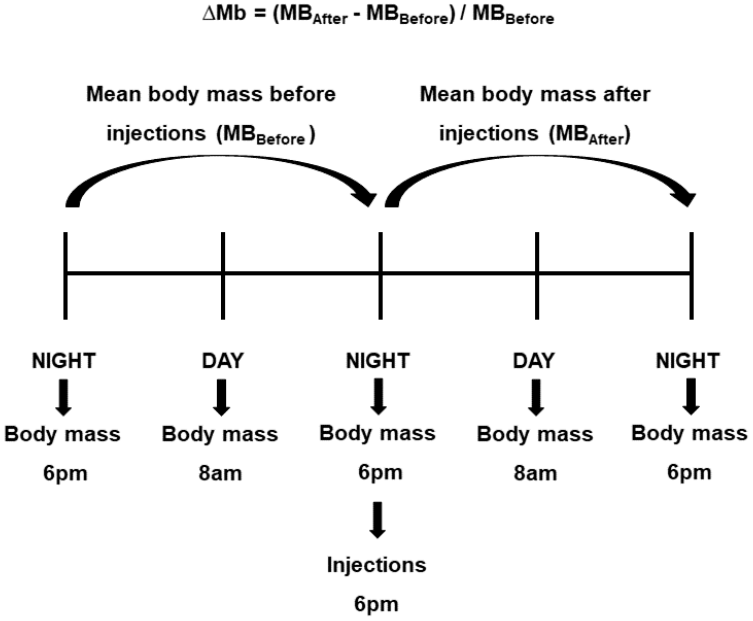

S1 Fig. Body mass change (ΔMb) of *C. perspicillata*. Body mass changes was assessed in relative terms as: body mass change (ΔMb) = (mean body mass after injections – mean body mass before injections) / (mean body mass before injections)
