# Supplementary material for: Combined effects of ambient temperature and food availability on induced innate immune response of a fruit-eating bat (Carollia perspicillata)
Source: PLoS One. 2024 May 24;19(5):e0301083. doi: 10.1371/journal.pone.0301083 (PMC11125493; doi:10.1371/journal.pone.0301083)
Supplement: S2 Fig — Hourly body temperature change was assessed 11 h after injections in absolute terms by subtracting hourly skin temperature after injections (TAhourly) from the respective hourly skin temperature before injections (TBhourly). (PDF) [file pone.0301083.s002.pdf]

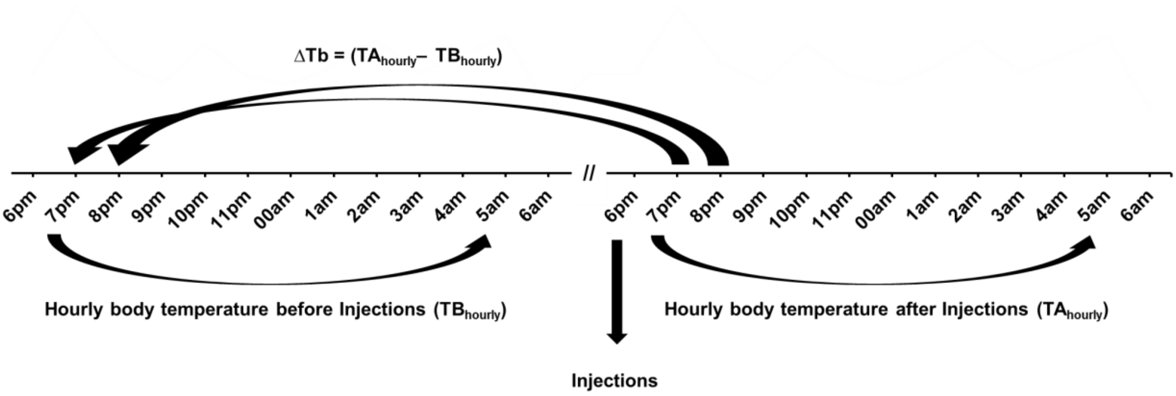

S2 Fig. Body temperature change (ΔTb) of *C. perspicillata*. Hourly body temperature change was assessed 11 h after injections in absolute terms by subtracting hourly skin temperature after injections (TA_{hourly}) from the respective hourly skin temperature before injections (TB_{hourly})
