# Supplementary material for: Combined effects of ambient temperature and food availability on induced innate immune response of a fruit-eating bat (Carollia perspicillata)
Source: PLoS One. 2024 May 24;19(5):e0301083. doi: 10.1371/journal.pone.0301083 (PMC11125493; doi:10.1371/journal.pone.0301083)
Supplement: S1 Table — Factorial ANOVA analyses was used to examine the effect of dose (PBS and 10 mg/kg LPS), ambient temperature (27°C and 33°C), diet (ad libitum feeding and restricted feeding) and the effects of their interactions on food intake change (ΔFI), body mass change (ΔMb), bacterial killing ability change (ΔBKA), total white blood cell change (ΔWBC) and neutrophil/lymphocyte ratio change (ΔNL). (PDF) [file pone.0301083.s003.pdf]

S1 Table. Result of analyzes for acute phase response components and bacterial killing ability in *Carollia perspicillata*. Factorial ANOVA analyses was used to examine the effect of dose (PBS and 10 mg/kg LPS), ambient temperature (27°C and 33°C), diet (ad libitum feeding and restricted feeding) and the effects of their interactions on food intake change (ΔFI), body mass change (ΔMb), bacterial killing ability change (ΔBKA), total white blood cell change (ΔWBC) and neutrophil/lymphocyte ratio change (ΔNL).

Variables	Factors	gl	F	Sig.	η^2
Food intake Change (ΔFI)	Dose	1	73.253	**<0.001**	0.567
	Feeding Regime	1	20.793	**<0.001**	0.271
	Ambient Temperature	1	2.980	0.090	0.051
	Dose*Feeding Regime	1	0.206	0.652	0.004
	Dose*Ambient Temperature	1	1.229	0.272	0.021
	Feeding Regime*Ambient Temperature	1	0.045	0.832	0.001
	Dose*Feeding Regime*Ambient Temperature	1	1.728	0.194	0.03
	Error	56			
Body mass Change (ΔMb)	Dose	1	56.713	**<0.001**	0.503
	Feeding Regime	1	25.31	**<0.001**	0.311
	Ambient Temperature	1	0.426	0.517	0.008
	Dose*Feeding Regime	1	0.001	0.98	0,000
	Dose*Ambient Temperature	1	0.134	0.716	0.002
	Feeding Regime*Ambient Temperature	1	0.069	0.794	0.001
	Dose*Feeding Regime*Ambient Temperature	1	0.025	0.874	0,000
	Error	56			
BKA Change (ΔBKA)	Dose	1	0.206	0.653	0.005
	Feeding Regime	1	0.568	0.455	0.014
	Ambient Temperature	1	0.28	0.599	0.007
	Dose*Feeding Regime	1	1.23	0.274	0.03
	Dose*Ambient Temperature	1	0.023	0.879	0.001
	Feeding Regime*Ambient Temperature	1	0.258	0.614	0.006
	Dose*Feeding Regime*Ambient Temperature	1	0.481	0.492	0.012
	Error	40			
WBC Change (ΔWBC)	Dose	1	1.375	0.246	0.024
	Feeding Regime	1	0.029	0.864	0.001
	Ambient Temperature	1	0.082	0.776	0.001
	Dose*Feeding Regime	1	0.020	0.887	0,000
	Dose*Ambient Temperature	1	0.688	0.41	0.012
	Feeding Regime*Ambient Temperature	1	0.209	0.649	0.004
	Dose*Feeding Regime*Ambient Temperature	1	0.295	0.589	0.005
	Error	56			
N/L Change (ΔNL)	Dose	1	157.691	**<0.001**	0.741
	Feeding Regime	1	7.789	**0.007**	0.124
	Ambient Temperature	1	4.792	**0.033**	0.08
	Dose*Feeding Regime	1	1.338	0.252	0.024
	Dose*Ambient Temperature	1	8.801	**0.004**	0.138
	Feeding Regime*Ambient Temperature	1	1.850	0.179	0.033
	Dose*Feeding Regime*Ambient Temperature	1	8.488	**0.005**	0.134
	Error	56			
