# Supplementary material for: Combined effects of ambient temperature and food availability on induced innate immune response of a fruit-eating bat (Carollia perspicillata)
Source: PLoS One. 2024 May 24;19(5):e0301083. doi: 10.1371/journal.pone.0301083 (PMC11125493; doi:10.1371/journal.pone.0301083)
Supplement: S2 Table — FIB: mean food intake before injections; FIA: mean food intake after injections; ΔFI: mean food intake changes after injections; WBCB: mean total white blood cell count before injections; WBCA: mean total white blood cell count after injections; ΔWBC: mean total white blood cell count change after injections; RNLB: mean neutrophil/lymphocyte ratio before injections; RNLA: mean neutrophil/lymphocyte ratio after injections; ΔRNL: mean neutrophil/lymphocyte ratio change after injections. BMB: mean body mass before injections; BMA: mean body mass after injections; ΔMb: mean body mass change after injections. N = 7 for ΔRNL of food restricted group at 27°C injected with LPS. N = 8 for all other groups. (PDF) [file pone.0301083.s004.pdf]

S2 Table. Changes in acute phase response components and bacterial killing ability of plasma after immune challenge in *Carollia perspicillata*

Temperature	Feeding Regime	Treatments	FIB	FIA	ΔFIA (%)	BMB	BMA	ΔMb (%)	BKAB	BKAA	ΔBKA (%)	WBCB	WBCA	ΔWBC (%)	RNLB	RNLA	ΔRNL (%)
27°C	Ad Libitum	PBS	17.63 ± 2.93	16.66 ± 3.47	-5.12 ± 13.69	13.87 ± 0.85	14.21 ± 1.09	-1.22 ± 2.71	66.91 ± 6.74	65.99 ± 7.54	-0.75 ± 13.49	4.14 ± 1.45	4.14 ± 1.15	2.18 ± 14.57	0.53 ± 0.13	0.54 ± 0.15	5.73 ± 35.94
		LPS	15.28 ± 2.46	4.41 ± 2.62	-69.89 ± 18.47	14.68 ± 1.04	13.54 ± 1	-6.81 ± 2.38	70.92 ± 6.44	75.5 ± 5.98	5.41 ± 8.15	4.26 ± 1.26	5.17 ± 2.2	20.11 ± 36.4	0.55 ± 0.13	1.57 ± 0.3	200.44 ± 88.77
	Resticted	PBS	6.96 ± 0.69	12.38 ± 4.82	79.97 ± 74.09	14.38 ± 1.25	14.16 ± 0.93	2.31 ± 2.96	64.04 ± 13.62	72.38 ± 10.86	9.65 ± 17.65	4.14 ± 1.45	3.78 ± 1.25	4.79 ± 19.93	0.58 ± 0.13	0.58 ± 0.12	3.33 ± 24.3
		LPS	7.26 ± 0.41	4.4 ± 3.96	-39.7 ± 53.41	14.53 ± 0.72	14.23 ± 0.83	-3.02 ± 4.36	65.24 ± 20.62	69.42 ± 10.99	10.63 ± 28.58	3.76 ± 1.7	4.03 ± 1.33	11.57 ± 17.94	0.74 ± 0.25	1.37 ± 0.41	57 ± 33.10
33°C	Ad Libitum	PBS	10.76 ± 5.01	10.36 ± 4.48	12.86 ± 62.6	14.85 ± 1.33	15.04 ± 1.32	-1.85 ± 2.37	70.34 ± 12.11	61.18 ± 17.44	-4.09 ± 28.73	4.17 ± 1.8	4.65 ± 2.44	9.35 ± 23.41	0.48 ± 0.16	0.5 ± 0.07	11.12 ± 29.18
		LPS	9.16 ± 3.5	0.63 ± 0.6	-92.89 ± 5.94	14.63 ± 1.8	14.22 ± 1.73	-6.71 ± 2.46	62.53 ± 15.59	66.04 ± 15.61	8.93 ± 26.11	3.72 ± 1.04	3.98 ± 1.1	8.53 ± 19.96	0.63 ± 0.14	1.98 ± 0.63	232.46 ± 150.34
	Resticted	PBS	7.33 ± 0.32	11.9 ± 4.29	64.21 ± 63.28	15.33 ± 0.96	15.08 ± 1.02	1.53 ± 1.63	55.5 ± 15.21	64.41 ± 12.13	8.43 ± 17.33	4.17 ± 1.8	3.81 ± 1.71	9.55 ± 14.07	0.61 ± 0.16	0.51 ± 0.12	-10.2 ± 32.5
		LPS	7.24 ± 0.42	3.31 ± 2.68	-55.58 ± 34.87	15.24 ± 0.73	14.11 ± 0.73	-3.51 ± 2.45	58.25 ± 13.28	60.36 ± 9.65	-0.83 ± 22.49	3.86 ± 1.25	4.37 ± 1.32	16.59 ± 31.16	0.58 ± 0.22	1.75 ± 0.34	228.94 ± 107.48
Mean between temperature and feeding regime treatments combined		PBS	10.67 ± 5.16	12.83 ± 4.72	37.98 ± 65.92	14.61 ± 1.2	14.62 ± 1.13	0.19 ± 2.95	63.67 ± 13.07	65.99 ± 12.23	3.45 ± 18.93	3.87 ± 1.5	4.09 ± 1.67	6.47 ± 17.78	0.55 ± 0.15	0.53 ± 0.12	2.5 ± 30.32
		LPS	9.73 ± 3.92	3.19 ± 3.04	-64.51 ± 37.4	14.77 ± 1.14	14.03 ± 1.12	-5.01 ± 3.39	64.05 ± 14.93	68.14 ± 11.69	6.03 ± 21.69	3.9 ± 1.28	4.38 ± 1.55	14.2 ± 26.49	0.62 ± 0.2	1.67 ± 0.5	184.02 ± 122.54
Mean between temperature and injections treatments combined		Ad Libitum	13.21 ± 4.86	8.02 ± 6.86	-38.76 ± 54.75	14.87 ± 1.31	14.25 ± 1.36	-4.15 ± 3.56	67.65 ± 10.6	67.45 ± 12.67	2.51 ± 19.47	4.07 ± 1.36	4.48 ± 1.8	10.04 ± 24.57	0.54 ± 0.15	1.15 ± 0.74	112.44 ± 136.64
		Resticted	7.2 ± 0.48	8 ± 5.7	12.23 ± 82.62	14.51 ± 0.97	14.4 ± 0.93	-0.67 ± 3.91	60.58 ± 15.61	66.64 ± 11.24	6.97 ± 21.03	3.7 ± 1.41	4 ± 1.36	10.63 ± 21.09	0.63 ± 0.2	1.05 ± 0.62	70.21 ± 113.69

FIB: mean food intake before injections; **FIA**: mean food intake after injections; **ΔFI**: mean food intake changes after injections; **WBCB**: mean total white blood cell count before injections; **WBCA**: mean white blood cell count after injections; **ΔWBC**: mean total white blood cell count change after injections; R**NLB**: mean neutrophil/lymphocyte ratio before injections; R**NLA**: mean neutrophil/lymphocyte ratio after injections; **ΔRNL**: mean neutrophil/lymphocyte ratio change after injections. **BMB**: mean body mass before injections; **BMA**: mean body mass after injections; **ΔMb**: mean body mass change after injections. N=7 for **ΔRNL** of food restricted group at 27°C injected with LPS. N= 8 for all other groups.
