# Supplementary material for: Combined effects of ambient temperature and food availability on induced innate immune response of a fruit-eating bat (Carollia perspicillata)
Source: PLoS One. 2024 May 24;19(5):e0301083. doi: 10.1371/journal.pone.0301083 (PMC11125493; doi:10.1371/journal.pone.0301083)
Supplement: S3 Table — Factorial ANOVA analysis was used to examine the effect of dose (PBS and 10 mg/kg LPS), ambient temperature (27°C and 33°C), feeding regime (ad libitum and food restricted) and the effects of their interactions on body temperature change. (PDF) [file pone.0301083.s005.pdf]

S3 Table. Results of body temperature analyzes in *Carollia perspicillata*. Factorial ANOVA analysis was used to examine the effect of dose (PBS and 10 mg/kg LPS), ambient temperature (27°C and 33°C), feeding regime (ad libitum and food restricted) and the effects of their interactions on body temperature change (ΔTb) in *Carollia perspicillata*

Variables	Factors	gl	F	Sig.	η^2
Body temperature Change (ΔTb)	Time	5	5.708	**<0.001**	0.093
	Time*Feeding Regime	5	1.490	0.197	0.025
	Time*Ambient Temperature	5	1.790	0.121	0.030
	Time*Dose	10	2.803	**0.002**	0.048
	Time*Feeding Regime*Ambient Temperature	5	1.607	0.164	0.027
	Time*Feeding Regime*Dose	5	1.093	0.363	0.019
	Time*Ambient Temperature*Dose	5	1.050	0.386	0.018
	Time*Feeding Regime*Ambient Temperature*Dose	5	0.637	0.658	0.011
	Error	258.33			
	Feeding Regime	1	0.213	0.645	0.003
	Ambient Temperature	1	1.739	0.192	0.030
	Dose	1	0.721	0.399	0.012
	Feeding Regime*Ambient Temperature	1	0.311	0.579	0.006
	Feeding Regime*Dose	1	3.018	0.087	0.051
	Ambient Temperature*Dose	1	0.451	0.504	0.007
	Feeding Regime*Ambient Temperature*Dose	1	0.992	0.323	0.017
	Error	56	0.001	0.985	0.000
Time to reached the maximum increase in ΔTb	Feeding Regime	1	4.079	**0.050**	0.127
	Ambient Temperature	1	1.751	0.196	0.058
	Feeding Regime*Ambient Temperature	1	0.819	0.373	0.028
	Error	28			

***** A factorial mixed ANOVA (3 Between subject factors / 1Within subject factor) was used to test effects of dose, ambient temperature, feeding Regime (as between subject factors), time (ΔTb for 11 hours as within subject factors) and the effects of their interactions on body temperature change (ΔTb). ** A factorial ANOVA was used to test effects of ambient temperature (27°C and 33°C) and feeding Regime (ad libitum and food restricted) and their interactions on the time at which the LPS-challenged groups reached the maximum increase in ΔTb.
