# Supplementary material for: Combined effects of ambient temperature and food availability on induced innate immune response of a fruit-eating bat (Carollia perspicillata)
Source: PLoS One. 2024 May 24;19(5):e0301083. doi: 10.1371/journal.pone.0301083 (PMC11125493; doi:10.1371/journal.pone.0301083)
Supplement: S4 Table — Hourly body temperature changes (ΔTb) after immune challenge in Carollia perspicillata kept at different ambient temperatures (27° and 33°C) and feeding regimes (ad libitum and food restricted). (PDF) [file pone.0301083.s006.pdf]

S4 Table. Body temperature changes in *Carollia perspicillata*. Hourly body temperature changes (ΔTb) after immune challenge in *Carollia perspicillata* kept at different ambient temperatures (27° and 33°C) and feeding regimes (ad libitum and food restricted). Data are given for all ambient temperature and feeding diets treatments combined.

Temperature	Feeding Regime	Treatments	1h	2h	3h	4h	5h	6h	7h	8h	9h	10h	11h
27°C	Ad Libitum	PBS	-0.47±0.38	-0.21±0.4	-0.13±0.38	-0.14±0.19	-0.11±0.41	-0.18±0.3	-0.13±0.2	-0.1±0.42	-0.1±0.42	-0.23±0.44	-0.24±0.39
		LPS	-0.5±0.29	-0.04±0.25	0.23±0.45	0.36±0.6	0.22±0.8	0.23±0.64	0.12±0.55	0.13±0.54	-0.03±0.49	-0.22±0.42	-0.26±0.56
	Resticted	PBS	-0.11±0.17	-0.02±0.35	-0.01±0.22	0.06±0.29	0.03±0.24	0.01±0.29	0.06±0.38	0.27±0.49	0.43±0.56	0.44±0.69	0.63±1.07
		LPS	-0.41±0.47	-0.31±0.44	0.13±0.59	0.48±0.46	0.53±0.39	0.65±0.19	0.29±0.39	0.52±0.56	0.26±0.48	0.35±0.66	0.24±0.92
33°C	Ad Libitum	PBS	-0.38±0.52	0.07±0.36	-0.11±0.62	-0.16±0.19	0.2±0.27	0.21±0.25	0.07±0.51	0.04±0.41	0.04±0.26	-0.03±0.28	-0.07±0.57
		LPS	-0.06±0.73	-0.17±0.57	-0.03±0.99	0.36±0.78	0.38±0.65	0.12±0.56	0.02±0.72	-0.1±0.49	-0.16±0.75	-0.02±0.65	-0.2±0.6
	Resticted	PBS	0.07±0.65	0.04±0.46	0.13±0.57	0.03±0.3	0.04±0.29	0.09±0.24	0.03±0.26	-0.04±0.64	0.08±0.34	-0.11±0.66	-0.18±1.12
		LPS	-0,14±0,43	-0,43±0,47	-0,29±0,29	-0,11±0,49	-0,01±0,87	0,12±0,55	0,22±0,58	0,19±0,61	0,04±0,89	-0,25±1,22	-0,34±1,2
Mean between temperature and feeding regime treatments combined		PBS	-0.22±0.49	-0.03±0.39	-0.03±0.47	-0.05±0.26	0.04±0.32	0.03±0.29	0.01±0.35	0.05±0.5	0.11±0.44	0.02±0.58	0.04±0.88
		LPS	-0.28±0.52	-0.24±0.45	0.01±0.63	0.27±0.61	0.28±0.7	0.28±0.53	0.16±0.55	0.18±0.57	0.03±0.66	-0.04±0.79	-0.14±0.85
