# Supplementary material for: Combined effects of ambient temperature and food availability on induced innate immune response of a fruit-eating bat (Carollia perspicillata)
Source: PLoS One. 2024 May 24;19(5):e0301083. doi: 10.1371/journal.pone.0301083 (PMC11125493; doi:10.1371/journal.pone.0301083)
Supplement: S5 Table — Time at which the LPS-challenged groups reached the maximum increase in ΔTb. (PDF) [file pone.0301083.s007.pdf]

S5 Table. Time of maximum increase of ΔTb in *Carollia perspicillata*. Time at which the LPS-challenged groups reached the maximum increase in ΔTb

Temperature	Feeding Regime	Time (h)
27°C	Ad Libitum	3.75±1.28
	Resticted	5.75±2.25
33°C	Ad Libitum	6.38±2.97
	Resticted	6.75±3.20
Mean between temperature treatments combined	Ad Libitum	4.75±2.04
	Resticted	6.56±2.99
