# Supplementary material for: Combined effects of ambient temperature and food availability on induced innate immune response of a fruit-eating bat (Carollia perspicillata)
Source: PLoS One. 2024 May 24;19(5):e0301083. doi: 10.1371/journal.pone.0301083 (PMC11125493; doi:10.1371/journal.pone.0301083)
Supplement: Attachment — Submitted filename: Response to Reviewers.pdf [file pone.0301083.s008.pdf]

Dear Editor

Thanks very much for your insightful comments. Below we replied to all points, and we hope that our responses and changes helped to clarify your concerns and improving the manuscript.

1 - Could the authors detail why they only considered the effect of changes in ambient temperature and food restriction for only two days?

AU: We kept the bats for 48 hours under these conditions because we did not want to compromise bat wellbeing; preliminary work with frugivorous bats showed that longer periods under temperature and feeding extreme conditions might be too demanding. Additionally, we were interested in short-period stress, a condition that might be common in nature.

2 - Why the authors analyzed all the parameters only 24 hours following LPS injection? Some parameters of the innate immune response and acute phase response will surely change within that window, but others regarding the adaptive immune response might take longer.

AU: This is an interesting idea, and we are aware that longer periods might show different patterns in elements of the immune response, but we were concerned that exposing bats to extreme experiment conditions for longer periods might be detrimental for their health. In our new version, we hope to have made our study goal clearer (line 120 to 139) to justify our approach.

3 - The authors indicate that they observed differences in the time to reach the highest increase in body temperature under food restriction and the magnitude of increased neutrophil/lymphocyte ratio under food restriction and increased ambient temperature.

A - Do these two parameters affect the immune performance when bats are challenged with real pathogens?

AU: Again, this is an interesting point worth examining in further studies. To the best of our knowledge, no previous bat study has tested the effect of manipulating ambient temperature and food availability in bat immune response to real pathogens. We hope that future studies explore this idea.

B - Is it possible to perform cell-culture experiments? Is it possible to measure serum antibodies or the antiviral response in bats?

AU: Yes, it is possible. Cell culture, serum antibodies and antiviral response have been done in bats (Dejosez et al. 2023; Banerjee et al. 2020). However, this idea goes beyond our study goals. In our new version, we hope to have made our object of study clearer (line 119 to 138).

Dejosez et al. 2023; https://doi.org/10.1016/j.cell.2023.01.011
Banerjee et al. 2020; https://doi.org/10.3389/fimmu.2020.00026

4 - The excessive use of abbreviations in the manuscript makes it very difficult to read

AU: Thanks for this comment, the excessive use of abbreviations in the text has been corrected, and we kept a few that we deemed would facilitate reading the manuscript.

5 - In line 50, the sentence has an extra punctuation

AU: Thanks, extra punctuation has been removed

6 - In line 121, please revise the sentence, as it says "In bats, recent reviews suggested that bats respond selectively to LPS..."

AU: Thanks, the sentence has been corrected
